# The Role of Mental Well-Being and Perceived Parental Supportiveness in Adolescents’ Problematic Internet Use: Moderation Analysis

**DOI:** 10.2196/26203

**Published:** 2021-09-15

**Authors:** Juwon Hwang, Catalina L Toma

**Affiliations:** 1 School of Media and Strategic Communication Oklahoma State University Stillwater, OK United States; 2 Department of Communication Arts University of Wisconsin-Madison Madison, WI United States

**Keywords:** problematic internet use, PIU, subjective mental well-being, perceived parental supportiveness, adolescents, well-being, young adult, internet, mental health, support, parent, engagement, social media

## Abstract

**Background:**

Given the growing number of adolescents exhibiting problematic internet use (PIU) and experiencing its harmful consequences, it is important to examine the factors associated with PIU. Existing research has identified perceived parental supportiveness and adolescents’ subjective mental well-being as strong predictors of PIU. However, it is unknown how these factors work together in shaping adolescents’ engagement in PIU.

**Objective:**

This paper aimed to examine the role played by adolescents’ perception of parental supportiveness in conjunction with their subjective mental well-being in shaping their PIU.

**Methods:**

The study analyzed one of the Technology & Adolescent Mental Wellness (TAM) data sets that were collected from a nationally representative cross-sectional sample. Adolescents self-reported their internet use behavior, perceived parental supportiveness, and subjective mental well-being through an online research panel survey. Hierarchical linear regression analysis with an interaction term was performed.

**Results:**

A total of 4592 adolescents, aged 12 to 17 years, completed the survey. Adolescents reported a mean age of 14.61 (SD 1.68) and were 46.4% (2130/4592) female and 66.9% (3370/4592) White. Findings revealed that, controlling for adolescents’ demographics and social media use, higher levels of perceived parental supportiveness (β=–.285, *P*<.001) and higher levels of subjective mental well-being (β=–.079, *P*<.001) were associated with a lower likelihood of adolescent PIU. The moderation analysis showed that the negative association between perceived parental supportiveness and PIU was stronger when adolescents reported high (vs low) levels of mental well-being (β=–.191, *P*<.001).

**Conclusions:**

This study shows that perceived parental supportiveness was a stronger protective factor than adolescents’ mental well-being against PIU. The protective power of perceived parental supportiveness against PIU was strongest when adolescents had high mental well-being. The highest risk of PIU occurred when adolescents’ mental well-being was high, but parents were perceived as unsupportive. Our findings suggest that parental supportiveness should be targeted as part of PIU prevention efforts.

## Introduction

### Background

Internet use has become a major part of adolescents’ daily life. A recent survey indicates that 45% of US adolescents aged 13 to 17 years are on the internet almost constantly [1], a figure that has nearly doubled from the 24% who reported being online on a near-constant basis in 2014-2015 [2]. Moderate internet use can be beneficial to adolescent development by facilitating social connectedness, providing useful information and entertainment, and helping with instrumental tasks (eg, [3,4]). However, excessive internet use can cause serious side effects, such as physical impairment, interpersonal problems, and poor academic performance [5,6].

A growing literature has conceptualized excessive internet use that leads to negative consequences in users’ lives as problematic internet use (PIU) [6,7]. Despite some disagreement on terminology and defining criteria [8], PIU is generally described as users’ excessive preoccupation with and loss of control over their internet use, resulting in negative personal and professional consequences [6,7]. Internet use refers to accessing the internet for information, entertainment, social connectedness, or other purposes using any device [9].

Adolescence is a particularly vulnerable period for the onset of PIU [10] because adolescents tend to exhibit lower levels of self-regulation [11]. Given the rewarding nature of internet use, low self-regulation is associated with increased risk for PIU [11-15]. In fact, one study found that PIU is more likely to occur in adolescents than in any other population [16]. Adolescent PIU is especially detrimental because it is likely to co-occur with other risky behaviors such as alcohol and drug use [10]. Thus, untreated adolescent PIU may transform into serious internet addiction in adulthood [15]. Given the prevalence and adverse outcomes of PIU among adolescents, it is important to identify the factors associated with PIU in this population. Below, we detail how two key factors—perceived parental supportiveness and adolescents’ subjective well-being—are expected to shape the development of adolescent PIU, separately and jointly.

### Perceived Parental Supportiveness and PIU

Adolescents are embedded into a family system that exercises tremendous influence over their lives. Thus, an extensive literature has examined adolescent PIU in the context of family interactions, especially parental supportiveness. Parental supportiveness is defined as “the extent to which parents intentionally foster individuality, self-regulation, and self-assertion by being attuned, supportive, and acquiescent to children’s special needs and demands” ([17]; see also Eastin et al [18]). Supportive parents provide meaningful explanations when setting limits and prohibitions on behavior, as well as unconditional positive regard for the child even when behavior does not match parents’ expectations or desires [19]. As a result, parental supportiveness helps children internalize and embrace their parents’ rules and values, thereby engaging in more prosocial behaviors [20,21]. Parental supportiveness has been shown to be key in promoting adolescents’ healthy social behaviors, such as volunteering and donating [21], and decreasing their problematic behaviors, such as cyberbullying [20] and affiliating with a deviant peer [22].

In the context of PIU, studies with US, European, and Asian samples show that supportive parenting practices, as indicated by high parent-child cohesion [23], high-quality parental relationships [24], parent-child bonding [25], and supportive parental monitoring [26], are protective factors against adolescent PIU. By the same token, unsupportive parental practices, as indicated by love withdrawal [27], authoritarian parenting style [28], high parent-child conflict [26], and rejecting, overprotective, or demanding parenting [28], substantially contribute to the development of adolescent PIU. Theoretically, the role played by parental supportiveness in the development of adolescent PIU has been explained through a compensation mechanism [29,30], whereby supportive parenting provides adolescents with a safe haven for sociopsychological development, but unsupportive parenting acts as a significant stressor, prompting adolescents to seek validation, support, and higher-quality relationships on the internet. In turn, adolescents’ reliance on the internet for compensating for deficits in parental supportiveness [29] can lead to overuse and compulsive use, the hallmarks of PIU [6-8]. An additional benefit of supportive parenting is that it improves adolescents’ emotional regulation skills, making it less likely that they will develop problems with impulse control [31,32].

An important note is that it is possible that unsupportive parents perceive themselves as supportive regardless of how the adolescents actually feel [33]. Since adolescents’ compensatory use of the internet is driven by their own perceptions of the family environment, this construct should be measured from the adolescents’ perspective. Hence, we hypothesize:

Hypothesis 1: Higher perceived parental supportiveness will be associated with lower PIU among adolescents.

### Subjective Mental Well-Being and PIU

Mental health can be conceptualized along two dimensions: psychopathology and subjective well-being [34]. Psychopathology refers to a severe disturbance in individuals’ actions, emotions, motivations, and cognitive and regulatory processes, causing distress and impairment in daily functioning [35]. Psychopathology is professionally diagnosed and includes disorders such as depression and anxiety. Subjective mental well-being, on the other hand, refers to the extent to which individuals experience optimal psychological functioning and a sense of thriving in their everyday life [36]. Subjective mental well-being includes an affective component (ie, the extent to which individuals experience positive, as opposed to negative, affect in everyday life) and a cognitive component (ie, the extent to which individuals are satisfied with their lives and feel agentic in tackling the challenges of everyday life) [36]. High subjective well-being is the result of high levels of positive affect, low levels of negative affect, and a high assessment of one’s own functioning.

While the two perspectives of mental health (ie, psychopathology and mental well-being) are related to each other, they are nonetheless distinct [34]. Simply put, the absence of mental illness does not mean individuals experience optimal psychological functioning and thriving. For example, it is possible that a nondepressed person (ie, absence of psychopathology) experiences low levels of positive affect in everyday life and may be dissatisfied with current life circumstances (ie, low subjective mental well-being).

An extensive literature has investigated the connections between adolescents’ mental health and their PIU, focusing primarily on psychopathology indicators such as depression and anxiety, and finding that they are significant risk factors for the development of adolescent PIU [12,37-40]. Recently, studies have turned their attention to dimensions of subjective mental well-being, given that these are more applicable to the broad population, as opposed to just clinical samples. Furthermore, PIU is considered a precursor to addiction, and therefore it is not itself an indicator of psychopathology. A similar pattern emerged across international samples, where high subjective mental well-being was robustly associated with low adolescent PIU [41-43], while indicators of low mental well-being, such as low self-esteem [14], self-control [15], and life satisfaction [13], were linked with high adolescent PIU.

High subjective mental well-being is theorized to act as a protective factor against the development of adolescent PIU because high-functioning individuals have more adaptive coping skills when faced with the stressors of daily life and are therefore less likely to turn to the internet to alleviate negative affective states [44]. The experience of sustained positive emotions also enables adolescents to think and act in more flexible and efficient ways, creating a cascade that builds enduring resources, both psychological and interpersonal [45]. Among these resources are better emotional regulation and impulse control, which protect against problematic engagement with the internet [44]. Consistent with these arguments, we hypothesize:

Hypothesis 2: Higher subjective mental well-being will be associated with lower PIU among adolescents.

### Interaction Effects of Subjective Mental Well-Being and Perceived Parental Supportiveness on PIU

While parental supportiveness contributes to high mental well-being among adolescents [46-48], mental well-being is considered a more comprehensive construct that may be related to, but not limited to, parental supportiveness [49]. Indeed, adolescent mental well-being is linked with a variety of other factors, such as the quality of peer relationships [50], peer support [51], sibling relationships [52], teacher caring [53,54], and academic achievement [55]. For example, a social environment with supportive parents but with low-quality peer relationships tends to result in significant impoverishment in adolescents’ subjective mental well-being [49].

Both parental supportiveness and mental well-being are expected to be protective factors against adolescent PIU, yet little is known about whether and how these factors work jointly in shaping PIU. We expect that the protective role of perceived parental supportiveness should be stronger for those adolescents with higher subjective mental well-being, since highly functioning adolescents more easily internalize the value and rules of positive social behaviors that their parents try to motivate [20,21]. Not only should adolescents high in subjective well-being be more responsive to parents’ supportiveness, thus eschewing PIU, but high parental supportiveness and high subjective well-being indicate a lack of significant stressors in daily life, which should also make it less likely for adolescents to turn to the internet in a compensatory manner. In other words, it is likely that the combination of high perceived parental supportiveness and high mental well-being is least likely to be associated with adolescent PIU.

On the other hand, those with low mental well-being and unsupportive parents should be especially vulnerable to PIU. As reviewed, unsupportive parenting may prompt adolescents to engage in excessive use of the internet [26-28], in an effort to compensate for an invalidating home environment [29,30]. Adolescents with low mental well-being may be more prone to engaging in this maladaptive practice because they lack good coping strategies to deal with stressful situations. Thus, it is likely that the strength of the negative association between parental supportiveness and PIU is weaker among those with low mental well-being than those with high mental well-being. In other words, the combination of low parental supportiveness and low mental well-being should be associated with the highest levels of PIU among adolescents. Thus, we hypothesize:

Hypothesis 3: High subjective mental well-being will moderate the association between perceived parental supportiveness and PIU, such that the negative association between perceived parental supportiveness and PIU will be stronger for adolescents with high mental well-being.

## Methods

### Data Collection

The study analyzed one of the Technology & Adolescent Mental Wellness (TAM) data sets that were collected from a nationally representative cross-sectional sample administered by Qualtrics between March and April 2019. The primary purpose for the data collection was to understand parents’ and adolescents’ technology use and mental health. The target population was English-speaking US residents aged 12 to 17 years. We set the parameters for Qualtrics to recruit a sample consistent with the race/ethnicity composition of the US census population for 12- to 17–year-old subjects. Recruitment and sampling approaches were modeled after previous youth and media studies using Qualtrics [56,57]. This study reports on data provided by adolescents, with the exception of two socioeconomic variables—family income and family structure—which were reported by their parents or guardians. This study was reviewed and approved by the Institutional Review Board at the University of Wisconsin-Madison.

### Measures

#### Problematic Internet Use

Adolescents completed the short version of the Problematic and Risky Internet Use Screening Scale (PRIUSS-3) [58]. The PRIUSS-3 was developed based on the PIU conceptual framework [6] and validated for use among adolescents and young adults [58], with strong reliability [59]. The PRIUSS-3 includes the following items: “how often do you experience increased social anxiety due to your internet use?,” “how often do you feel withdrawal when away from the internet?,” and “how often do you lose motivation to do other things that need to get done because of the internet?,” scored on a 5-point Likert scale from 0 (never) to 4 (very often). Items were summed to create a PIU score for each participant, ranging from 0 to 12 (mean 4.72, SD 3.50; Cronbach α=.87).

#### Perceived Parental Supportiveness

Adolescents answered the following questions about their relationship with their parent or guardian who took the survey with them using a 5-point Likert scale from 0 (never) to 4 (always) [46]: “how often does she/he praise you for doing well?,” “how often does she/he criticize you or your ideas?,” “how often does she/he help you do things that are important to you?,” “how often does she/he blame you for her/his problems?,” and “how often does she/he make plans with you and cancel for no good reason?.” The questionnaire demonstrated good reliability in previous research [46]. Items were recoded to indicate higher values as higher supportiveness. We removed an item (“how often does she/he criticize you or your ideas?”), which caused weak reliability. Responses were summed to create a score for each participant, ranging from 0 to 16 (mean 12.38, SD 3.2; Cronbach α=.79).

#### Subjective Mental Well-Being

The short version of the Warwick-Edinburgh Mental Well-Being Scale (SWEMWBS) was used to measure adolescents’ mental well-being (7 items) [60]. The WEMWBS is a measure of mental well-being focusing entirely on positive aspects of mental health and shows strong criterion and content validity [60]. Adolescents reported how they felt in the past 2 weeks about the following statements on a scale from 1 (none of the time) to 5 (all of the time): “I’ve been feeling optimistic about the future,” “I’ve been feeling useful,” “I’ve been feeling relaxed,” “I’ve been dealing with problems well,” “I’ve been thinking clearly,” and “I’ve been able to make up my own mind about things.” Responses were summed to produce a score for each participant, ranging from 1 to 30 (mean 22.73, SD 4.35; Cronbach α=.83).

### Socioeconomic and Social Media Use Variables

Age, gender, race, family income, family structure, school type, and social media use were included as covariates in the analysis. Respondents were asked to indicate their age, ranging from 12 to 17 years. Gender was coded with 1 being female and 2 being male. Race was coded with 1 being Caucasian and 0 being others. School type was categorized into 1 being public schools and 0 being others. Family income was assessed using 12 increasing income ranges (1=less than $20,000 to 12=more than $150,000). Family structure was coded with 1 being a parent who is divorced, separated, or widowed and 0 being others. Finally, frequency of checking social media was assessed on a scale from 1 (less than once a week) to 8 (almost constantly) [61].

### Analytic Strategy

The hypotheses were tested through a hierarchical linear regression analysis conducted using the *lmSupport* package in R (R Core Team). Perceived parental supportiveness and subjective mental well-being were entered as independent variables and PIU as a dependent variable. We controlled for age, gender, race, family income, family structure, school type, and the frequency of checking social media. Predictors were mean-centered before they were entered in the moderated regression model. Multicollinearity was not an issue, with the variance inflation factor statistic for the predictors ranging from 0.52 to 1.93.

## Results

A total of 4592 parent-adolescent (aged 12-17 years) dyads completed the survey. Adolescents’ mean age was 14.61 (SD 1.68) years, and the sample consisted of 46.4% (n=2130) females and 66.9% (n=3370) White individuals. Table 1 presents more descriptive information. Bivariate Pearson correlations among all variables included in this analysis are presented in Table 2.

Most caregivers identified themselves as a biological parent (n=3934, 85.7%), followed by stepparent (n=246, 5.4%), parent’s partner (living together) (n=137, 3.0%), adoptive parent (n=120, 2.6%), grandparent (n=106, 2.3%), other relative or guardian (n=26, 0.6%), and foster parent (n=12, 0.3%).

**Table 1:** Descriptive characteristics (N=4592).

Characteristic		Participants
Age (years), mean (SD)		14.61 (1.68)
**Gender, n (%)**		
	Female	2130 (46.4)
	Male	2392 (52.1)
	Nonbinary gender	23 (0.5)
	Female-to-male transgender	25 (0.5)
	Male-to-female transgender	5 (0.1)
	Prefer not to answer	17 (0.4)
**Race/ethnicity, n (%)**		
	White/Caucasian	3370 (66.9)
	Black or African American	699 (15.2)
	American Indian/Alaska Native	116 (2.5)
	Asian	211 (4.5)
	Asian Indian	17 (0.4)
	Other Asian	7 (0.2)
	Native Hawaiian/other Pacific Islander	36 (0.8)
	Multiracial	221 (4.8)
	Other	31 (0.7)
	Prefer not to answer	82 (1.8)
	Latino/Hispanic/Mexican	101 (2.2)
**Family income (US$)^a^, n (%)**		
	Less than $9,999	235 (5.1)
	$10,000-$19,999	310 (6.8)
	$20,000-$29,999	417 (9.1)
	$30,000-$39,999	441 (9.6)
	$40,000-$49,999	385 (8.4)
	$50,000-$59,999	440 (9.6)
	$60,000-$69,999	306 (6.7)
	$70,000-$79,999	399 (8.7)
	$80,000-$89,999	266 (5.8)
	$90,000-$99,999	322 (7.0)
	$100,000-$149,999	694 (15.1)
	More than $150,000	368 (8.0)
**Family structure^a^, n (%)**		
	Married	2921 (63.6)
	Living with a partner	440 (9.6)
	Divorced	389 (8.5)
	Separated	136 (3.0)
	Widowed	94 (2.0)
	Never married	568 (12.4)
	Prefer not to answer	44 (1.0)
**School type, n (%)**		
	Public school (middle or high school)	3585 (78.1)
	Private school (middle or high school)	568 (12.4)
	Home schooled	207 (4.5)
	Online school	106 (2.3)
	Public 4-year college	82 (1.8)
	Not currently in school	23 (0.5)
	Prefer not to answer	19 (0.4)
**Social media use, mean (SD)**		
	Frequency of checking social media	5.15 (2.06)

^a^This item was answered by parents or guardians who took the survey with the adolescents.

**Table 2:** Pearson correlation coefficients for all variables.

Variables	1	2	3	4	5	6	7	8	9	10
1. Age	—^a^	—	—	—	—	—	—	—	—	—
2. Gender	–0.021	—	—	—	—	—	—	—	—	—
3. Race	0.059^b^	0.065^b^	—	—	—	—	—	—	—	—
4. Family income	0.026	0.115^b^	0.160^b^	—	—	—	—	—	—	—
5. Family structure	0.052^b^	–0.032^d^	–0.024	–0.218^b^	—	—	—	—	—	—
6. School type	0.007	–0.058^b^	–0.049^c^	–0.102^b^	–0.041^c^	—	—	—	—	—
7. Frequency of checking social media	0.047^c^	–0.048^e^	0.038^d^	0.059^b^	–0.069^b^	–0.069^b^	—	—	—	—
8. Mental well-being	–0.010	0.047^c^	0.046^c^	0.114^c^	–0.036^d^	–0.004	–0.005	—	—	—
9. Parental supportiveness	0.057^b^	–0.081^b^	0.011	–0.069^b^	0.079^b^	0.152^b^	–0.146^b^	0.228^b^	—	—
10. Problematic internet use	–0.045^c^	0.040^c^	0.083^b^	0.106^b^	–0.066^b^	–0.140^b^	0.353^b^	–0.104^b^	–0.366^b^	—

^a^The correlation coefficient is not displayed since it is shown in the asymmetrically diagonal position of the table.

^b^Correlations significant at the *P=*.001 level.

^c^Correlations significant at the *P=*.01 level.

^d^Correlations significant at the *P=*.05 level.

Standardized coefficients, standard errors, and *P* values for the independent variables and all covariates are summarized in Table 3. PIU was associated with being younger (β=–.072, *P*<.001), being White (β=.053, *P*<.001), having a higher family income (β=.050, *P*=.002), attending nonpublic school (β=–.060, *P*<.001), and using social media more often (β=.277, *P*<.001).

There was a statistically significant negative relationship between perceived parental supportiveness and PIU (β=–.275, *P*<.001), meaning that adolescents who perceived their parents as more supportive were less likely to engage in PIU. Thus, hypothesis 1 was supported. Similarly, there was a statistically significant negative association between subjective mental well-being and PIU (β=–.079, *P*<.001), meaning that adolescents with higher levels of subjective mental well-being were less likely to be engaged in PIU. Thus, hypothesis 2 was supported.

A statistically significant interaction effect of perceived parental supportiveness and subjective well-being on PIU also emerged (β=–.191, *P*<.001), meaning that the protective power of perceived parental supportiveness against PIU was strongest when adolescents had high mental well-being, supporting hypothesis 3.

**Figure 1:**
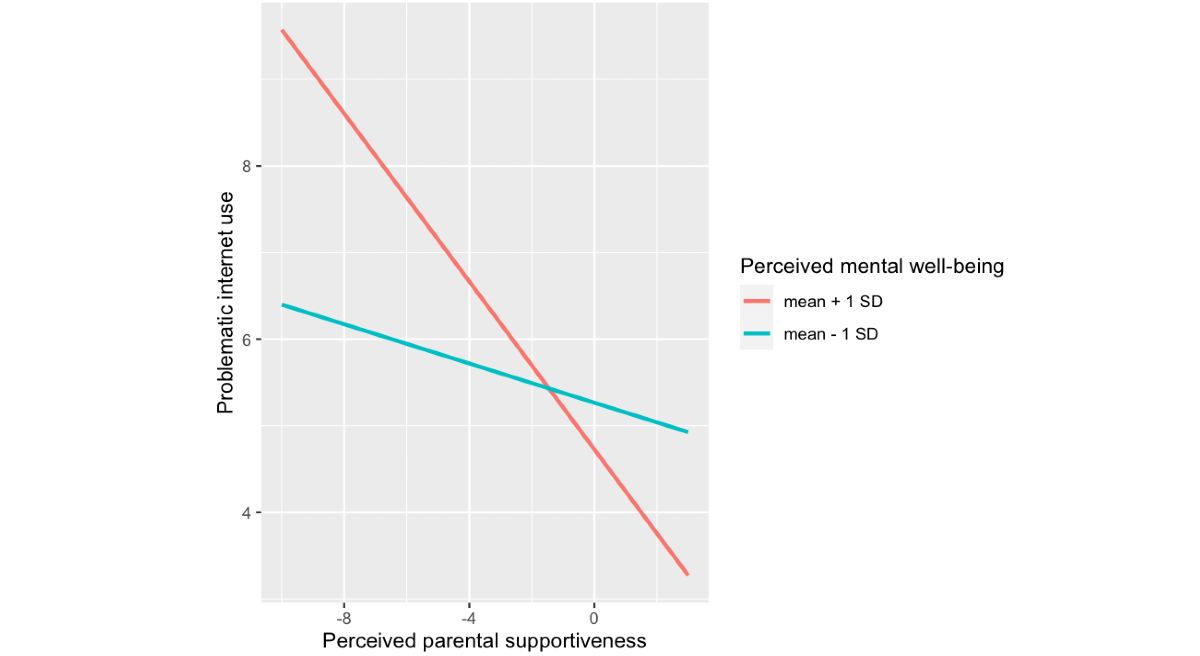
Interaction effect between perceived parental supportiveness and subjective mental well-being on problematic internet use.

Simple slope analyses for the association between perceived parental supportiveness and PIU were calculated at the mean (1 SD) level for subjective mental well-being, using the Johnson-Neyman techniques [62]. At mean – 1 SD of subjective mental well-being, the slope was b=–.12, SE 0.02, *t*=–4.98, *P*<.001. At mean + 1 SD of subjective mental well-being, the slope was b=–.49, SE 0.02, *t*=–20.88, *P*<.001. This revealed that the negative association between perceived parental supportiveness and PIU was significantly stronger for those with high subjective mental well-being than for those with low subjective mental well-being (Figure 1). Thus, the protective power of perceived parental supportiveness against PIU was highest when adolescents had high mental well-being.

**Table 3:** Hierarchical regression analysis examining the relationships between problematic internet use, perceived parental supportiveness, and subjective mental well-being (N=4592)^a^.

Variable		Problematic internet use		*P* value	Δ*R*^2^ (%) (total *R*^2^=28.5%)
		β	SE		
**Control variables**					22.2
	Age	–.072	0.032	<.001	
	Gender^b^	.002	0.107	.90	
	Race^c^	.053	0.115	<.001	
	Family income	.050	0.016	.002	
	Family structure^d^	–.020	0.160	.22	
	School type^e^	–.060	0.135	<.001	
	Frequency of checking social media	.277	0.027	<.001	
**Independent variables**					4.2
	Perceived parental supportiveness	–.285	0.018	<.001	
	Subjective mental well-being	–.079	0.011	<.001	
**Interactions**					2.2
	Perceived parental supportiveness × subjective mental well-being	–.191	0.003	<.001	

^a^All coefficients are standardized. Predictors are mean-centered.

^b^Female=1, male=2.

^c^White=1, others=0.

^d^Divorced, separated, or widowed parent=1, others=0.

^e^Public school=1, others=0.

## Discussion

### Principal Findings

PIU among adolescents is a public health concern due to its high prevalence and detrimental impact on adolescents’ physical, social, and academic development [5,6]. The goal of this study was to examine the role played by two psychological factors in the development of adolescent PIU—perceived parental supportiveness and subjective mental well-being—through a large, nationally representative survey of US adolescents aged 12 to 17 years.

Our main findings can be summarized as follows: the more adolescents perceived their parents as supportive, the less PIU they reported, consistent with research that shows adolescents who perceive their parents as supportive internalize rules and values for prosocial behavior and do not need to resort to internet use to compensate for deficits in parental supportiveness. Similarly, better mental well-being among adolescents was associated with lower PIU, supporting claims by previous research [44,45] that high-functioning individuals have more adaptive coping skills, better emotional regulation, and more internal resources for coping with everyday stressors, without turning to the internet as a refuge or distraction. Notably, the effect of perceived parental supportiveness on PIU was quite large (β=–.278, *P*<.001) and substantially larger than the effect of subjective mental well-being on PIU (β=–.071, *P*<.001), indicating that perceived parental supportiveness may be a key driver of PIU in adolescents, and thus merits attention in future research.

Finally, the negative association between PIU and perceived parental supportiveness increased for adolescents with high mental well-being (Figure 1). PIU was lowest among adolescents with supportive parents and high mental well-being, which indicates that, unsurprisingly, those who are well adjusted and come from supportive environments are protected against risky behaviors such as PIU. However, an unexpected result emerged: PIU was highest among those with unsupportive parents and *high* mental well-being. While we do not have data on either adolescents’ motivations for spending excessive amounts of time on the internet or on the specific activities they undertake online, we have argued that adolescents who perceive their parents as unsupportive turn to the internet to find solace, companionship, and understanding, consistent with the compensation mechanism articulated by prior research [29,30]. This finding suggests that it is highly functioning adolescents who are more likely to engage in this compensatory use of the internet. Those who have higher mental well-being may be better attuned to the opportunities provided by the internet to compensate for face-to-face deficits—in our case, a lack of parental supportiveness—and more agentic in pursuing those opportunities. Unfortunately, however, the more time they spend online, likely communicating with peers or seeking other opportunities for fun and relaxation (eg, video games), the more likely they are to rely on the internet to a problematic extent.

We conducted additional analyses to investigate the possibility that adolescents who perceive their parents as unsupportive seek social connections on the internet, potentially in an effort to foster relationships that compensate for low parental supportiveness. A regression model with social media use as the dependent variable and parental supportiveness and mental well-being as independent variables (Multimedia Appendix 1) confirmed that social media use was highest among those with unsupportive parents and high mental well-being. Since social media is a venue for fostering social connections, primarily with peers, this finding provides support for the social compensation hypothesis, whereby teens who perceive their parents as unsupportive parents go online to seek more meaningful social connections (see also Anderson and Jiang [1] and Barker [30]). Initially, this compensatory internet use may be an adaptive practice, but the rewarding nature of the internet can prompt increasing use and overreliance, putting adolescents on a slippery slope toward PIU and later on even internet addiction.

An intriguing issue that should be investigated by future research concerns the interplay between PIU/internet addiction and subjective mental well-being over time. While adolescents with unsupportive parents in our sample maintained high levels of subjective mental well-being even as they engaged in high PIU, it is likely that as PIU continues over time it can lead to a deterioration in subjective mental well-being, and even to psychopathology, supporting the large body of research that finds depression and anxiety to be strongly linked with PIU and internet addiction in young adults [12,37-40]. Although most studies to date are cross-sectional and treat PIU as the outcome of mental health indicators, it is likely that the causal relationship between PIU and mental health is bidirectional, with both variables influencing each other over time. Thus, it is possible that high levels of PIU will, in turn, negatively impact adolescents’ mental well-being in the long run.

Given this state of affairs, we argue that adolescence is a tremendously important point for intervention, before PIU turns into full-blown internet addiction and before it leads to a deterioration in adolescents’ well-being. Perceptions of parental unsupportiveness were the strongest driver of problematic usage, even among adolescents who otherwise experienced high well-being. Thus, perceived parental supportiveness is a key factor that should be targeted. For example, PIU prevention should include training for parents to improve their communication skills and provide appropriate discipline, but also validation and companionship, for their adolescents.

### Limitations and Future Directions

This study has several limitations. While associations were observed between PIU and subjective mental well-being and perceived parental supportiveness, the cross-sectional nature of the study does not permit insight into temporal or causal relationships. Future longitudinal studies are necessary to understand what factors protect adolescents against PIU. This study did not differentiate between diverse types of internet use (eg, video gaming, chatting, social networking sites, etc). PIU in these different online contexts may relate differently to subjective mental well-being and perceived parental supportiveness. Furthermore, the measures are all self-reported by adolescents and thus are limited by their ability and willingness to recall and report information accurately.

### Conclusion

Despite these limitations, this study contributes to the literature by finding a meaningful interplay between adolescents’ mental well-being and their perception of parental supportiveness in shaping PIU. This helps illuminate the conditions under which adolescent PIU emerges.

## Appendix

Multimedia Appendix 1 Interaction effect between subjective mental well-being and perceived parental supportiveness on the frequency of checking social media.

## Abbreviations

PIU: problematic internet use
PRIUSS-3: Problematic and Risky Internet Use Screening Scale
SWEMWBS: Warwick-Edinburgh Mental Well-Being Scale–short version
TAM: Technology & Adolescent Mental Wellness
WEMWBS: Warwick-Edinburgh Mental Well-Being
