# Supplementary material for: The Role of Mental Well-Being and Perceived Parental Supportiveness in Adolescents’ Problematic Internet Use: Moderation Analysis
Source: JMIR Ment Health. 2021 Sep 15;8(9):e26203. doi: 10.2196/26203 (PMC8550797; doi:10.2196/26203)
Supplement: Multimedia Appendix 1 [file mental_v8i9e26203_app1.docx]

**Appendix 1.** *Interaction effect between subjective mental well-being and perceived parental supportiveness on the frequency of checking social media*

**
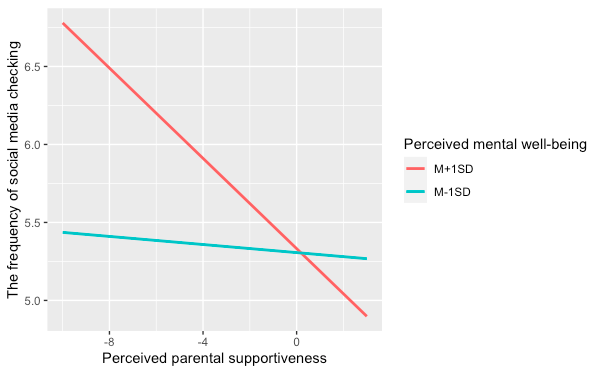
**
